# A Psychometric Analysis of Raven’s Colored Progressive Matrices: Evaluating Guessing and Carelessness Using the 4PL Item Response Theory Model

**DOI:** 10.3390/jintelligence10010006

**Published:** 2022-01-25

**Authors:** Faye Antoniou, Ghadah Alkhadim, Angeliki Mouzaki, Panagiotis Simos

**Affiliations:** 1 Department of Secondary Education, College of Education, National and Kapodistrian University of Athens, Panepistimioupolis, 15772 Zografou, Greece; 2 Department of Psychology, College of Arts, Taif University, P.O. Box 11099, Taif 21944, Saudi Arabia; ghadah.s@tu.edu.sa; 3 Department of Primary Education, University of Crete, 74100 Rethimno, Greece; angeliki.mouzaki@gmail.com; 4 School of Medicine, University of Crete, 71500 Giofirakia, Greece; akis.simos@gmail.com

**Keywords:** nonverbal IQ, Raven’s colored progressive matrices, item response theory, guessing, carelessness

## Abstract

The purpose of the present study was to evaluate the psychometric properties of Raven’s colored progressive matrices by estimating the presence of pseudo-guessing and pseudo-carelessness. Participants were 1127 children from ages 5 to 11. Guessing and carelessness were assessed using the lower and upper asymptotes of the 3PL and 4PL item response theory (IRT) models, respectively. Optimal model fit was judged using difference loglikelihood tests and information criteria. Results indicated that guessing, but not carelessness, were evident in the AB and B forms of the CPM, with successful guessing being more prevalent in the AB form. It is concluded that nonverbal IQ estimation in CPM should include variable estimation methods so that aptitude scores are estimated with the highest possible accuracy.

A valid assessment of ability is extremely important, as several decisions that affect one’s life are based on these evaluations (Facon et al. 2011). For example, success at school, entrance to university, placement in general or special education settings, success in the workplace, and social approval are some consequences of ability testing; thus, issues of equity and fairness should be prominent components of authorities’ educational policies (Zumbo 2007).

## 1. Raven’s Colored Progressive Matrices

The colored progressive matrices (CPM) were developed in 1938 and have been revised twice since then (1947 and 1956, Raven et al. 1998) for the purpose of evaluating fluid intelligence (Spearman’s g concept) in young children. Each item involves a visual matching task with six options in which a target stimulus is matched to a complex pattern. The measure was developed for ages 5–11, and since then it has been subject to several validation studies among English-speaking or international participants, which have produced strong evidence for its construct (Facon et al. 2011; Goharpey et al. 2013; Van Herwegen et al. 2011) and discriminant validity, such as when demonstrating cognitive dysfunction in cerebral palsy (Pueyo et al. 2008). The matrices have been normed in over 35 countries and are now considered to be one of the most valid measures of g (Jensen 1998) or general intelligence (Abad et al. 2004; Mackintosh 1996), especially as a culture-free measure. Interestingly, a Google search reveals 6,820,000 pages for CPM and google scholar shows 23,400 empirical studies, verifying their wide implementation and use. Physiological studies have also linked this measure of non-verbal skill with specific brain areas related to receptive language (Prabhakaran et al. 1997), as well as with well-known measures of receptive language skills (e.g., the PPVT, Kilburn et al. 1966). What is less known, however, is how the different styles of respondents tend to affect the estimation of that person’s ability and the validity of the inferences made. Among the factors that affect responding, guessing in multiple-choice formats and careless responding are two salient examiner behaviors that can distort measurement (Leong et al. 2013). The two factors are described below as a function of their estimation via the 3-PL and 4-PL item response theory (IRT) models (Barton and Lord 1981; Loken and Rulison 2010).

## 2. Item Response Theory Models

In the family of IRT models, the one-parameter logistic model posits that the probability of correctly responding to item *j* for person *i* is given by the expression (Waller and Reise 2009):

(1) $$P_{1\mathrm{PL}}\left(Y_{ij}=1|b_{i},\theta_{j}\right)=\frac{e^{\left(\theta_{j}-b_{i}\right)}}{1+e^{\left(\theta_{j}-b_{i}\right)}}$$

where the probability of correctly responding to item *i* is a function of person *j*’s ability *θ* and the item’s difficulty level *b_i_*. The term *e* = 2.71828. The one-parameter model only manipulates the ability of a person, and assumes that the relationship between the person’s ability and item difficulty is consistent across items (i.e., the item slopes are fixed to unity). Thus, the total score is a sufficient statistic for this model, despite the fact that different response patterns can result in the same total score. Birnbaum (1968) relaxed this assumption by allowing these relationships to vary across items (i.e., by allowing item characteristic curves—ICCs—to cross), thus enabling the estimation of different levels of discrimination *α*. Consequently, with the addition of the discrimination parameter α, the one-parameter model takes the following form and becomes the two-parameter IRT logistic model:

(2) $$P_{2\mathrm{PL}}\left(Y_{ij}=1|a_{i},\;b_{i},\theta_{j}\right)=\frac{e^{a_{i}\left(\theta_{j}-b_{i}\right)}}{1+e^{a_{i}\left(\theta_{j}-b_{i}\right)}}$$

where α estimates the degree to which an item discriminates between various levels of the latent trait theta, with steeper slopes associated with greater discrimination and vice versa.

With dichotomous items, the probability of correctly responding to an item ranges between 0 and 1 across all levels of ability *θ*. For polytomous measures, however, the probability rarely approaches zero, as for low abilities, guessing is associated with levels of ability that are greater than zero. Thus, the probability of correctly responding needs to be corrected for guessing, which is why the 3PL model has been proposed by Barton and Lord (1981):

(3) $$P_{3\mathrm{PL}}\left(Y_{ij}=1|a_{i},\;b_{i},c_{i},\theta_{j}\right)=c_{i}+\left(1-c_{i}\right)*\frac{e^{a_{i}\left(\theta_{j}-b_{i}\right)}}{1+e^{a_{i}\left(\theta_{j}-b_{i}\right)}}$$

which adds the pseudo-guessing parameter *c_i_* to assess the magnitude of correct responding for individuals with infinitely low ability levels (Waller and Reise 2009). The item guessing parameter *c_i_* is estimated as follows:

(4) $$c_{i}=\frac{e^{\left(\gamma_{i}\right)}}{1+e^{\left(\gamma_{i}\right)}}$$

with $\gamma_{i}$ being the second threshold of the 3PL model, reflecting the lower asymptote of the item characteristic curve (ICC).

Controlling for the lower asymptote accounts for guessing, but it is imperative to also account for individual differences in the probability that higher-ability individuals miss easy items due to carelessness, attention problems, socially desirable responding, distractions, or other influences, such as a lack of motivation to engage with an easy task (Liao et al. 2012). Thus, the four-parameter model has been proposed to account for these influences (Barton and Lord 1981) by allowing the upper asymptote to vary as follows (Waller and Feuerstahler 2017):

(5) $$P_{4\mathrm{PL}}\left(Y_{ij}=1|a_{i},\;b_{i},c_{i},d_{i}\theta_{j}\right)=c_{i}+\left(d_{i}-c_{i}\right)*\frac{e^{a_{i}\left(\theta_{j}-b_{i}\right)}}{1+e^{a_{i}\left(\theta_{j}-b_{i}\right)}}$$

The model adds the parameter d*i*, which indicates the magnitude of the upper asymptote. The item for the upper asymptote is estimated as follows:

(6) $$d_{i}=\frac{e^{\left(\delta_{i}\right)}}{1+e^{\left(\delta_{i}\right)}}$$

with $\delta_{i}$ being the second threshold of the 4PL model. Despite the premise of the 4PL as an alternative psychometric model, few evaluations are currently available (e.g., Liao et al. 2012; Reise and Waller 2003), limiting its generality as a useful analytical tool. However, recent simulation studies have confirmed that the 4PL model showed higher precision and a lower amount of bias compared to the 3PL model under computerized adaptive testing (CAT) (Liao et al. 2012) and under real and ideal data (Waller and Feuerstahler 2017).

In the context of the above four models, aberrant responding reflects model misfit and a discrepancy between the person’s observed and expected behavior, resulting in a violation from the model expectations. In item response models, a Guttman scaling pattern is expected, in which individuals are expected to be successful on items for which their ability is higher and unsuccessful in items that exceed their level of ability. For example, using a hypothetical 10-item scale, a person of average ability is expected to be successful in the first three items, have a 50% likelihood of success in the next four items, and is expected to fail in the last three items (i.e., have an expected response pattern: 1,1,1,0,1,0,1,0,0,0). For that average ability person, failing any of the first three items that are within their level of ability may be indicative of carelessness and would point to unexpected responding. Similarly, if that person is successful in any of the last three items for which their level of ability does not suffice, that would also be indicative of aberrant responding, e.g., in the form of a lucky guess, cheating, etc. By modeling these behaviors using the 3PL and 4PL item response models, an estimation of a person’s abilities may be more accurate, as the additional sensitivity will result in the higher precision and accuracy of person-based scores.

**Figure 1:**
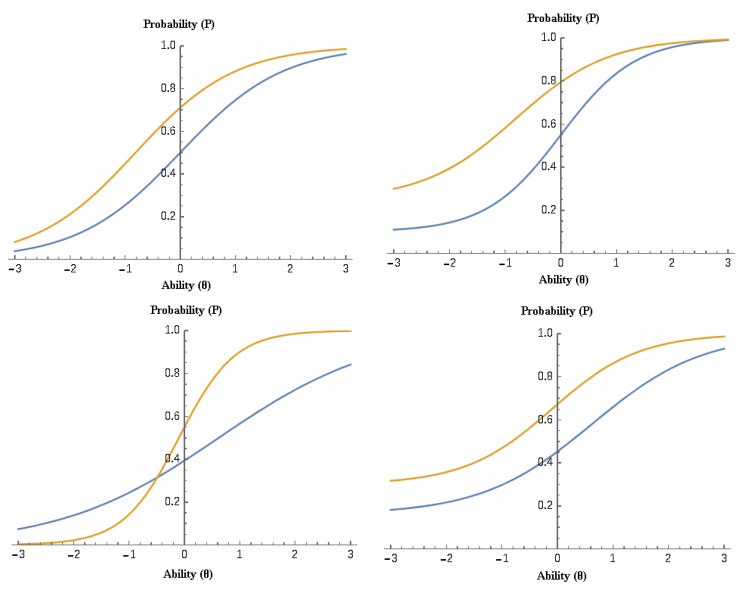
Item Characteristic Curves (ICCs) for two sample items (yellow and blue lines) per IRT model: 1PL (**top left**), 2PL (**bottom left**), 3PL (**top right**), and 4PL (**bottom right**).

Figure 1 displays the item characteristic curves (ICCs) for each of the four IRT models. In the top-left figure, the one-parameter model shows two items with the differences in ability levels required by equal discrimination (slopes). The bottom-left figure adds the discrimination parameter, showing a higher discriminating ability for the item with the steeper curve (the 2-PL model). The top-right figure shows two items with varying degrees of guessing, as the lower asymptote is non-zero (the 3-PL model). Finally, the bottom right figure shows non-zero upper asymptotes to control for carelessness, in addition to the estimation of the earlier mentioned parameters.

## 3. Importance of the Present Study

An enormous amount of effort has been dedicated to obtaining valid estimates of the latent traits of aptitude and ability. To this end, advances in modeling need to be employed to ensure that the estimates of person attributes are as accurate as possible. In multiple-option tests, one is bound to have some non-zero estimates of ability due to correctly guessing one among several options. Such estimates can greatly inflate a person’s scores and lead to invalid conclusions; thus, one must account for them. Similarly, careless mistakes due to anxiety or attention problems can greatly deflate a person’s scores and invalidate their estimates by heavily penalizing them based on these early misses (Mislevy and Bock 1982). The punitive effects of respondent carelessness have been previously reported regarding stress (Rulison and Loken 2009). Thus, the unbiased measurement of ability requires accounting for such response disturbances by implementing the 3-PL and 4-PL IRT models (Magis 2013). One purpose of the present study was to investigate the extent of these extraneous influences on score estimation, as decreased interest in these estimations, particularly those of carelessness, has been mostly due to difficulties in applying the models, complexity, and the accessibility of available software (Loken and Rulison 2010). A secondary purpose was to compare models that account for those influences (3-PL and 4-PL) with those not accounting for them (2-PL) to propose the best analytical strategy for the estimation of fluid intelligence using Raven’s CPM.

## 4. Method

### 4.1. Participants and Procedures

The sample comprised 1127 children from ages 5 to 11 and represented the country of Greece (530 boys, 597 girls). Students were selected using stratified random sampling procedures, with the strata being geographical area and parents’ education. This normative sample included 84 students with identified disabilities (e.g., 44 with LD and 30 with EMR). The rates of disability and parents’ education matched Greek census data. All available data were utilized, along with the small percentages of individuals with disabilities, as they were also representative of the population of interest.

The CPM was administered as a paper-and-pencil assessment at public schools during regular school hours and it took on average approximately 30 min to complete all three forms. The participating students were pulled out of their classrooms and were administered the CPM on a one-to-one basis. The children were told that the purpose of the study was to see how they think about geometric matching and that this ‘game’ would have no influence whatsoever on their school grades. They were also assured that they could withdraw their participation at any time and were given the test after ensuring that (a) they were in good physical condition and (b) understood the directions of the instrument. Informed consent was provided by students’ parents before the administration of the test.

As the smaller unit of analysis would involve analyses by age group, a power simulation model was run to ensure that 100 participants would suffice and help us to obtain stable parameters of ability. The model was simulated with averaged parameters derived from the sample. The results indicated that bias was minimal with the present sample sizes. The difference in RMSEA values was equal to 0.008 (i.e., expected 5%, estimated 5.8%). The probability of rejecting coefficients at 95% (for a nominal alpha level of 5%) ranged between 93.7% and 95.2%. Thus, the overall conclusion was that the power levels were sufficient to measure stable parameters per age group.

### 4.2. Measures

Raven’s CPM includes three forms of increasing difficulty (namely A, AB, and B), with each form consisting of 12 items for a total of 36 items. There is only one correct stimulus among six stimuli that the participant needs to match to a pre-specified pattern. Thus, a dichotomous response option was engaged.

### 4.3. Data Analyses

A series of IRT models from the Rasch through to the four-parameter models were fit to the data. The model comparison was based on the difference in loglikelihoods, which is distributed as a chi-square statistic along with an expanded list of information criteria. When moving from less to more parameterized models, the interest is in the 3PL and 4PL models. If the 3PL model was deemed superior compared to the 2PL model, then that would be evidence that successful guessing is operative. If the 4PL model is superior to the 3PL model, then that would indicate that both successful guessing and carelessness are likely operative. The results from the omnibus test statistics were judged considering the excessive power levels, given that the models were run with 1127 participants. Thus, even minimum discrepancies between observed and expected response patterns would be deemed significant through the use of the chi-square statistic; instead, more value was placed on the information criteria, and mostly on the Bayesian information criterion (BIC) and its small sample size variant (i.e., SABIC), as the Akaike information criterion (AIC, Akaike 1974) often favors larger models (i.e., heavily parameterized models). Additional information criteria were the approximate weight of evidence (AWE) criterion, the Hannan–Quinn (HQ) criterion and variates of AIC (for more information, see Masyn 2013), although other corrective procedures are also available (e.g., Vandekerckhove et al. 2015). For the information criteria, the difference in estimates between the adjacent models greater than 10 points was considered, favoring the model with the smaller value (Burnham and Anderson 2002). The test response functions (TRFs) and test information functions (TIFs) were plotted for each of the CPM’s forms to examine the sensitivity of the forms to different ability levels (*θ*). The level of significance was set to 1/1000 due to the excessive power of the inferential statistical tests. Given that pseudo-guessing and pseudo-carelessness are item-based parameters, as shown in Equations (4) and (6), we utilized an R function to estimate guessing and carelessness rates for each person. Those estimates were plotted and were also tested against the null hypothesis that their levels were zero and 1, respectively (for lower and upper asymptotes). All IRT models were run using Mplus 8.7 and were also replicated with IRTPro 4.2 (up to 3PL). Per-person estimates for guessing and carelessness were estimated using the R function irt4plpf (https://github.com/GS1968/CFAtools/blob/main/4PL_15.R, accessed on 16 January 2022). Each person’s aberrant response patterns were assessed using the R package PERFIT (Tendeiro et al. 2016). Among the various indices, the U_3_ (Van der Flier 1982), C (Sato 1975), and H_t_ (Sijtsma 1986) statistics were utilized, as they were found to behave better with brief instruments (Karabatsos 2003).

## 5. Results

### 5.1. Construct Validity between Forms A, AB, and B across 1-PL through to 4-PL IRT Models

Prior to testing the construct validity of the separate forms, it was important to establish that the three forms comprised distinct, albeit correlated, constructs. To this end, a unidimensional structure in which all items were loaded on a general intelligence factor was contrasted to a model that specified three distinct constructs (i.e., the forms). Results using information criteria indicated preference of the three-forms model against the unidimensional model (AIC_3Forms_ = 33,174.014, AIC_Uni_ = 33,241.893; BIC_3Forms_ = 33,732.046, BIC_Uni_ = 33,784.842, and the sample-adjusted BIC, that is the SABIC_3Forms_ = 33,739.479, SABIC_Uni_ = 33,441.804). Thus, utilizing separate forms and fitting models per form seems to be the most appropriate choice with the present national data. Table 1 displays model fit and difference statistics across the four IRT models and per each of the CPM forms. For forms A and B, more heavily parameterized models were favored compared to the more parsimonious models. Thus, the 4PL model was favored over the 3PL model. On the other hand, for form AB, the 3PL model provided a superior fit, suggesting the presence of significant guessing, but no careless responding. Thus, the omnibus test statistics suggested the presence of both guessing and carelessness (forms A and B) or guessing alone (form AB). These results, however, should be interpreted with caution, as excessive power likely governed the magnitude of the chi-square test statistics for which minimal discrepancies between the data and perfect model fit would be found to be significant given the sample size *n* > 1000. Thus, more attention was given to the information criteria and the distributions of each person’s guessing and carelessness parameters, which are discussed next.

**Figure 2:**
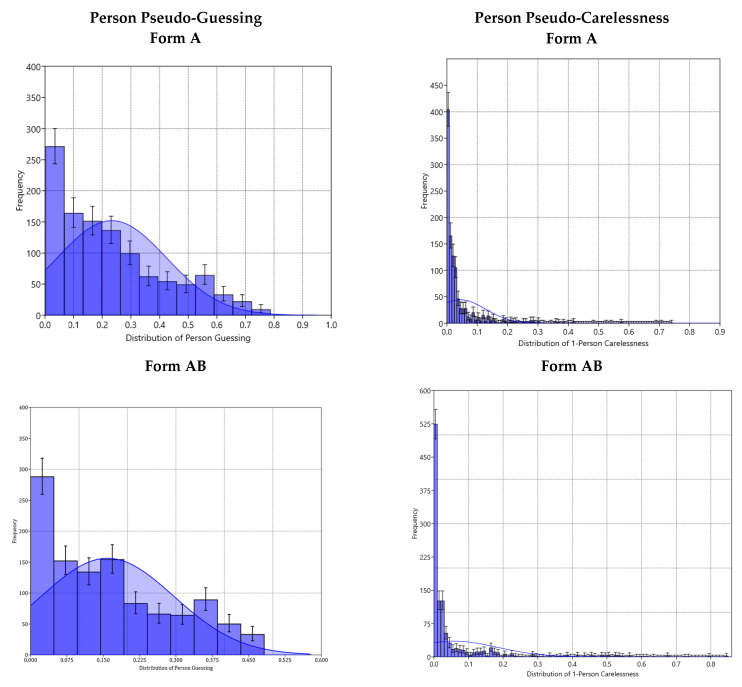

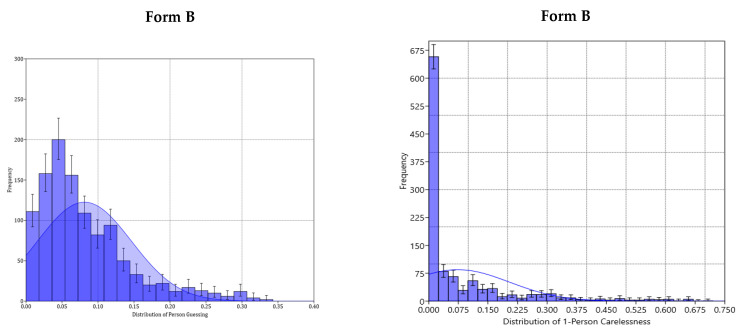
Distributions of person pseudo-guessing (left panel) and 1-pseudo-carelessness (right panel) for forms A (**top**), AB (**middle**), and B (**bottom**) for CPM.

Table 2 displays the information criteria to aid the evaluation of the optimal model fit. Among the information criteria, the literature favors the BIC and its variant, SABIC, more so compared to the Akaike-based indices, in which more parameterized models are given precedence (Burnham and Anderson 2002). As shown in the table, form A resulted in a very mixed picture, with CAIC and AWE favoring the Rasch model, BIC favoring a 2PL model, SABIC and HQ favoring the 3PL model, and AIC and HQ favoring the 4PL model. Thus, based on the information from the inferential statistics and information criteria, a solution for form A was not clearly favored, suggesting the need to complement the current analyses with additional information. After applying a function to estimate person-based guessing and carelessness behaviors, the results indicated that the mean estimate of pseudo-guessing in form A was equal to 0.233 (SD = 0.191; C.I._95%_: 0.009–0.679). This estimate is clearly higher than what was expected from random guessing (i.e., 1/6 = 16.7%), suggesting the presence of informed guessing, as the ability of the person likely sufficed to decipher “problems” for which the level of ability was not quite required; however, the importance of removing irrelevant distractors is evident, as that elimination process likely increased the levels of success to more than what was expected from chance alone. As shown in the upper-left panel of Figure 2, more than 75% off the participants displayed non-zero guessing behaviors. In contrast, pseudo-carelessness was not evident. As shown in the figure (in the upper-right panel) 1-pseudo-carelessness was close to zero. Specifically, the point estimate of carelessness was 0.044 (SD = 0.082; C.I._95%_: 0.001–0.289).

Given the preference of a 3PL model for the AB form using inferential statistics, the information criteria corroborated with the same conclusion, except for the AWE. Specifically, the AIC, BIC, SABIC, CAIC, AICc, and HQ agreed, and although the AWE favored a 2PL model, the difference in AWE values was less than 10 units, suggesting a least meaningful difference between models based on conventions governing information criteria (Raftery 1995). Thus, the 2PL model was not preferred. Further evidence in favor of a 3PL model and against a 4PL model was provided by plotting each person’s guessing and carelessness estimates (Figure 2). As shown in the figure (middle panel), the mean estimate of pseudo-guessing in form AB was equal to 0.164 (SD = 0.189; C.I._95%_: <0.001–0.677) and for pseudo-carelessness, it was equal to 0.056 (SD = 0.126; C.I._95%_: <0.001–0.499). Thus, the non-zero and approximately zero levels of pseudo-guessing and pseudo-carelessness estimates provided further support for concluding that the 3PL model was appropriate with this form.

Lastly, form B was also favored by the 3PL model, pointing to the presence of non-zero levels of guessing, but it failed to support the presence of significant levels of pseudo-careless responding. Among the information criteria, the 3PL model was favored by BIC, SABIC, CAIC, AWE, and HQ. Only AIC and its variant, AICc, favored a 4PL model. The mean estimate of pseudo-guessing in form B was equal to 0.093 (SD = 0.062; C.I._95%_: 0.007–0.247) and for pseudo-carelessness, it was equal to 0.064 (SD = 0.102; C.I._95%_: <0.001–0.377). Thus, the non-zero and approximately zero levels of pseudo-guessing and pseudo-carelessness estimates provided further support for concluding that the 3PL model was appropriate with this form. Thus, for reasons of parsimony, the 3PL model was deemed the most appropriate model with these data as well.

**Figure 3:**
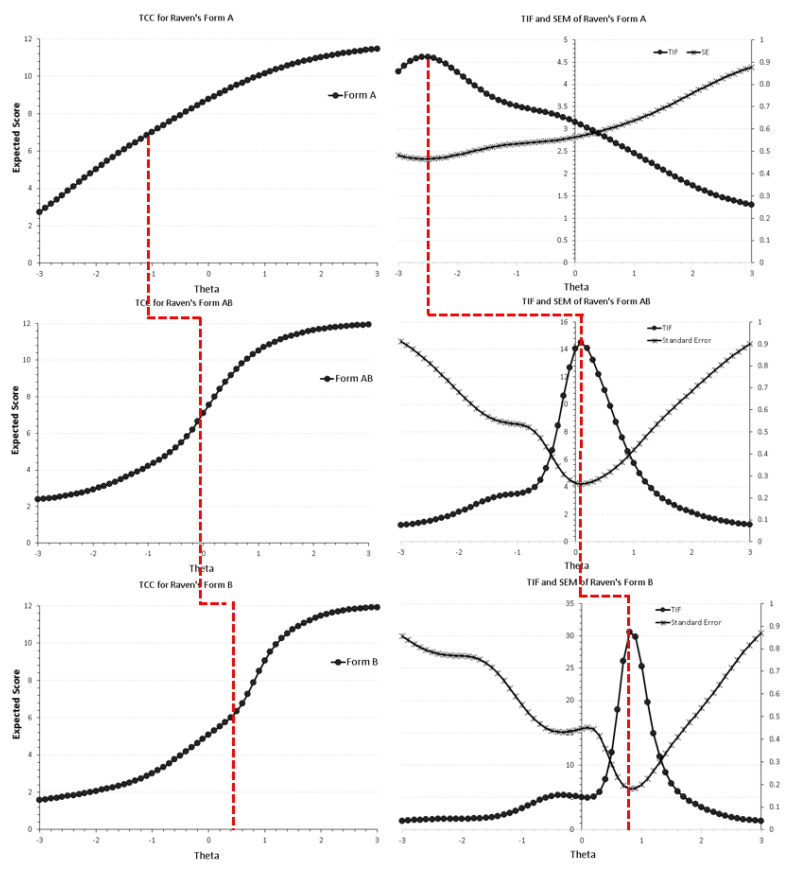
Test response functions (TRF, **left panel**) and test information functions (TIFs, **right panel**), along with conditional standard errors of measurement for forms A, AB, and B in Raven’s colored progressive matrices after fitting the optimal IRT model for each form (i.e., 3PL). The dashed vertical line indicates theta levels that are associated with success rates of 50%. The sensitivity of each form to different ability levels is evident.

Figure 3 displays the test response curves (TRC), test information functions (TIFs), and conditional standard errors of measurement (CSEs) for each of the three forms, using the 3PL model as a means of visually evaluating each form’s sensitivity to specific levels of theta. For example, for form A, the mean difficulty estimate was equal to −1.04 (SD = 1.80), suggesting that the A form is easy and required −1 logits in person ability. Moving from form A to form AB pointed to a difference of −0.95, as the mean difficulty level of the AB form was equal to −0.09 (SD = 0.99) or just average. Lastly, form B, designed to be the most difficult of all, had a mean of 0.21 (SD = 1.12) logits, suggesting the need for above-average ability levels for participants to be successful. Information curves for each form show that form A is sensitive to very low levels of theta and becomes much less sensitive at above-average levels; form AB shows the maximum information right around zero, showing maximum sensitivity for average ability individuals. Lastly, form B is more sensitive to above-average ability levels. The reciprocal of the information curves, that is, the conditional errors of measurement, mirror these results with respect to error per theta level.

### 5.2. An Example of Guessing and Carelessness: Plotting Person Response Functions

**Figure 4:**
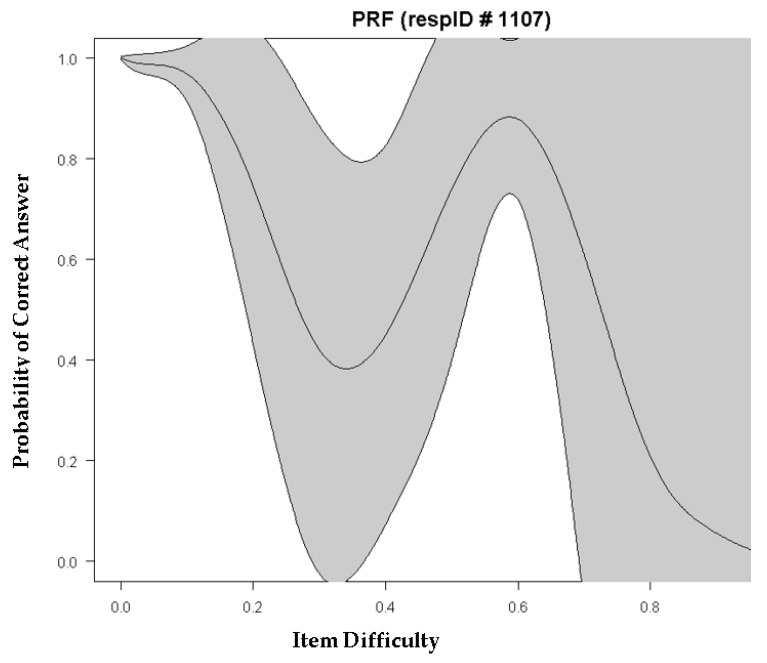
Person response function (PRF) for participant 1107, for which an aberrant response pattern is evident.

A more in-depth analysis of the occurrence of guessing was conducted for participant 1107, for which their response vector was as follows: 1,1,1,0,0,0,1,1,1,1,1,0. The maximum likelihood estimate of theta for that person was −0.230, and their personal guessing estimate was 24%. However, what is interesting is that this person fails items 4, 5, and 6, which are much easier compared to items 7 through 11, which are much harder, but the person succeeds on them. This behavior is shown in Figure 4, where a negative linear trajectory should govern the relationship between the probability of success and item difficulty. However, as shown below, items in the difficulty range between 0.4 and 0.7 have a higher probability of success compared to easier items (at the 0.3 difficulty level). This aberrant response pattern likely represents some type of guessing behavior (lucky or informed), as participant 1107 does not appear to have the requisite skills to be successful in those difficult items. The classification of participant 1107 as a guesser is further confirmed using the U_3_, C, and H_t_ statistics, for which the cutoff values are >0.25, >0.53, and <0.22, respectively, all indicating a violation from the perfect Guttman pattern (Karabatsos 2003). The estimates of participant 1107 on these statistics were as follows: U_3_ = 0.302, C = 0.724, and H_t_ = 0.169, far above or below the cutoff values that maximized sensitivity and specificity in the Karabatsos (2003) simulation study.

Interestingly, participant 1107 could also serve as an example of careless responding. If we assume that success at levels of difficulty of 0.6 logits are not lucky guesses, but rather reflect true skill, then the failures taking place between 0.3 and 0.4 logits likely represent careless responding.

## 6. Discussion

The purpose of the present study was to evaluate the psychometric properties of Raven’s colored progressive matrices by estimating the presence of pseudo-guessing and pseudo-carelessness in the estimation of non-verbal aptitude.

The most important finding of the present study was that non-zero person parameters of guessing were observed with regard to Raven’s colored progressive matrices. When comparing nested models and penalizing the one estimating a larger number of parameters (i.e., the 4-PL), the findings indicated a preference for the 4PL model. However, this preference was attributed to excessive levels of power for the omnibus chi-square test. Using the information criteria results indicated a preference for the 3PL model, suggesting non-zero levels of pseudo-guessing or “informed” guessing. Informed guessing is involved when individuals utilize prior knowledge to eliminate erroneous distractors, and although requisite knowledge is lacking, they end up selecting among maybe two options; thus, successful guessing probability is highly increased (from 1/6, to 1/2). This success is obviously more prevalent for individuals of higher ability or generally those who encounter items for which their level of ability or skill suffices. Thus, for accurately estimating a person’s nonverbal intelligence via the CPM, the 3-PL model is expected to provide the most unbiased estimates of a person’s skill and should be the preferred choice for all forms. This proposition disagrees with the recommendations put forth by Waller and Reise (2009) for the measurement of psychopathology who, although they failed to report superior model fit for the 4-PL model, nevertheless recommended it because it was associated with very different information functions compared to the respective functions of the 3-PL model. The Waller and Reise (2009) recommendations may partly depend on the observation that non-zero levels of carelessness are present in the estimation of various attributes. In the present study, carelessness levels were not prevalent, but its levels were also non-zero. In fact, they represented small to medium effect sizes, but were nevertheless small in absolute value terms (around 5–6%). This finding may suggest that systematic sources of measurement error, such as fatigue, may likely be operative. These estimates cannot be attributed to item content and socially desirable responding, as suggested earlier when measuring attitudes and sensitive domains (Osgood et al. 2002), as the matrices represent a non-verbal mean of assessing intelligence.

The investigation of person response functions, such as the example provided for participant 1107, provide useful information for understanding the pattern of aberrant responding. In the present example, it was evident that participant 1107 was a low-achieving person with a negative logit on theta and was nevertheless successful in some difficult items, despite missing some earlier items. Guessing was evident using a person pseudo-guessing estimate of 0.24, which is far above zero levels or even levels of lucky guessing (i.e., 1/6 possibilities). Thus, this person likely engaged in informed guessing, or even cheating. In other words, we can only speculate what the cause is for aberrant responding, although we can clearly identify the individuals who reflect unexpected response patterns, such as participant 1107. In those instances, the use of the 3PL model can help to identify such individuals, and decisions can be made, e.g., (a) for them to sit the test again, (b) to exclude the participant from the normative sample, etc.

The third important finding was that the order of difficulty between forms was validated. As shown via the TRFs and TIFs, forms A, AB, and B are of increasing difficulty, covering with increased accuracy specific levels of theta. Specifically, form A is sensitive for below-average ability levels, form AB for average ability individuals, and form B for those with above-average ability. This finding agrees with a series of studies in which the CPM proved to possess construct validity across various populations (ethnic and/or minority groups) under specific conditions (e.g., low threat; see Brown and Day 2006).

Practically speaking, identifying the most appropriate measurement model using item response models contributes to obtaining the most accurate measurement for a person’s skills and competencies. IRT models have increased sensitivity through appropriately weighing the contribution of each item to the latent construct as well as item difficulties. Thus, they move away from the crude approximation of number-correct scores provided in classical test theory (CTT). Furthermore, using IRT models’ information related to aberrant responding, such as unexpected successes (e.g., lucky guessing, copying, and cheating) or unexpected failures (such as careless responding, being inattentive or unmotivated), can be modelled rather than ignored, contributing to a more accurate estimate of person measurement on a given latent trait.

The present study is limited for several reasons. First, some difficulty in the estimation of upper and lower asymptotes has been reported due to requirements for specific constraints on other parameters and their magnitude (Linacre 2004; Rupp 2003), which, over time, has contributed to the unpopularity of the models. Concerns over the reliability of estimating the asymptotes using maximum likelihood methods have also been raised earlier (Baker and Kim 2004). Recent advances in software, however, and corrections to known problems have been proposed (e.g., Magis 2013; Swaminathan and Gifford 1986). Second, the findings can only be generalized to the population of Greek children, as normative findings between countries have indicated salient differences in ability (Raven et al. 1998). Third, we did not test for specific age X gender interactions, although such findings have been reported in the literature (Savage-McGlynn 2012) but were beyond the focal research question of the present study.

In the future, it will be important to evaluate various properties of the CPM with regard to multi-population invariance and predictive validity. Furthermore, the contribution of the newly included vocabulary test to the measurement of crystallized intelligence needs to be ascertained, along with its relationship to ‘g’. Last, identifying whether a partial credit model would be psychometrically sound is another novel direction for research.

## Figures and Tables

**Table 1:** Comparison between Rasch, 2-PL, 3PL, and 4PL IRT models using omnibus test statistical criteria per form of CPM.

Models Tested	LL	Npar	scf	Model Comparison	LRTS	Dtsc/d.d.f.	Chi-Square
Form A							
M1. Rasch Model	−5067.533	12	0.981	-	-	-	-
M2. 2-PL IRT Model	−5019.974	24	1.118	M1 vs. M2	95.118	1.255/12	75.791 ***
M3. 3-PL IRT Model	−4985.443	36	1.146	M2 vs. M3	69.062	1.202/12	57.456 ***
M4. 4-PL IRT Model	−4970.947	48	1.044	M3 vs. M4	28.992	0.738/12	39.285 ***
Form AB							
M1. Rasch Model	−6856.604	12	0.958	-	-	-	-
M2. 2-PL IRT Model	−6646.828	24	1.043	M1 vs. M2	419.552	1.128/12	371.943 ***
M3. 3-PL IRT Model	−6547.913	36	1.055	M2 vs. M3	197.830	1.079/12	183.346 ***
M4. 4-PL IRT Model	−6540.517	48	20.585	M3 vs. M4	14.792	79.175/12	0.187
Form B							
M1. Rasch Model	−6336.923	12	0.941	-	-	-	-
M2. 2-PL IRT Model	−6108.592	24	1.079	M1 vs. M2	456.662	1.217/12	375.236 ***
M3. 3-PL IRT Model	−5971.200	36	1.001	M2 vs. M3	274.784	0.845/12	325.188 ***
M4. 4-PL IRT Model	−5957.052	48	1	M3 vs. M4	28.296	0.997/12	28.381 **

Note: LL = loglikelihood; Npar = number of estimated parameters; scf = scaling correction factor; LRTS = likelihood ratio statistic; dtsc = difference test scaling correction factor; d.d.f. = difference in degrees of freedom. *** *p* < 0.001; ** *p* < 0.01.

**Table 2:** Comparison between Rasch, 2PL, 3PL, and 4PL IRT models using information criteria ^†^ per form of CPM.

Model Tested	AIC	BIC	SABIC	CAIC	AWE	AICc	HQ
Form A							
M1. Rasch Model	10,159.066	10,219.394	10,181.278	**10,231.394**	**10,339.722**	10,159.35	10,181.86
M2. 2-PL IRT Model	10,087.948	**10,208.604**	10,132.373	10,232.604	10,449.259	10,089.04	10,133.54
M3. 3-PL IRT Model	10,042.886	10,223.869	**10,109.523**	10,259.869	10,584.853	10,045.33	**10,111.27**
M4. 4-PL IRT Model	**10,037.894**	10,279.205	10,126.744	10,327.205	10,760.516	**10,042.26**	10,129.08
Form AB							
M1. Rasch Model	13,737.208	13,797.536	13,759.420	13,809.536	13,917.864	13,737.49	13,760.00
M2. 2-PL IRT Model	13,341.656	13,462.312	13,386.081	13,486.312	**13,702.967**	13,342.74	13,387.25
**M3. 3-PL IRT Model**	**13,167.826**	**13,348.809**	**13,234.463**	**13,384.809**	13,709.793	**13,170.27**	**13,236.21**
M4. 4-PL IRT Model	13,177.034	13,418.345	13,265.884	13,466.345	13,899.656	13,181.40	13,268.22
Form B							
M1. Rasch Model	12,697.846	12,758.174	12,720.058	12,770.174	12,878.502	12,698.13	12,720.64
M2. 2-PL IRT Model	12,265.184	12,385.840	12,309.609	12,409.840	12,626.495	12,266.27	12,310.77
**M3. 3-PL IRT Model**	12,014.400	**12,195.383**	**12,081.037**	**12,231.383**	**12,556.367**	12,016.84	**12,082.79**
M4. 4-PL IRT Model	**12,010.104**	12,251.415	12,098.954	12,299.415	12,732.726	**12,014.47**	12,101.29

Note: AIC = Akaike information criterion; BIC = Bayesian information criterion; SABIC = sample-adjusted BIC; CAIC = consistent AIC; AWE = approximate weight of evidence criterion; AICc = corrected AIC; HQ = Hannan–Quinn criterion. Smaller values of the information criteria are indicative of a better model fit. Estimates in bold show model preference for a given information criterion. ^†^ Difference estimates greater than 10 units are considered to be meaningful, favoring models with smaller information criteria values (Burnham and Anderson 2002).

## Data Availability

Data are in a repository at the publisher Motivo, Topos as they were part of the standardization of Raven’s CPM in the Greek population.
